# Screening of *BRCA1/2* variants in Mauritanian breast cancer patients

**DOI:** 10.1186/s12885-022-09903-8

**Published:** 2022-07-20

**Authors:** Selma Mohamed Brahim, Ekht Elbenina Zein, Crystel Bonnet, Cheikh Tijani Hamed, Malak Salame, Mohamed Vall Zein, Meriem Khyatti, Ahmedou Tolba, Ahmed Houmeida

**Affiliations:** 1 Unité de Recherche sur les Biomarqueurs dans la Population Mauritanienne. UNA-FST. Unité URBPM Nouakchott-Mauritanie, Nouakchott-Mauritanie, France; 2 Centre National d’Oncologie (CNO). Unité de Recherche et d’Enseignement, Nouakchott-Mauritanie, France; 3 Institut de l’Audition, Institut Pasteur, Inserm, Paris, France; 4 Centre d’Hépatovirologie, Nouakchott-Mauritanie, France; 5 Institut Pasteur du Maroc. Laboratoire Oncologie et Thérapie Cellulaire, Casablanca -aroc, Morocco

**Keywords:** Breast cancer (BC), *BRCA1/2*, Variant, Women, Mauritania

## Abstract

**Background and study aim:**

Carrying a pathogenic *BRCA1/2* variant increases greatly young women’s risk of developing breast cancer (BC). This study aimed to provide the first genetic data on BC in Mauritania.

**Methods:**

Using NGS based screening; we searched for *BRCA1/2* variants in DNA samples from 137 patients diagnosed for hereditary BC.

**Results:**

We identified 16 pathogenic or likely pathogenic (PV) variants carried by 38 patients. Two predominant *BRCA1* PV variants were found: c.815_824dup and c.4986 + 6 T > C in 13 and 7 patients, respectively. Interestingly, three novels *BRCA1/2* predicted pathogenic variants have also been detected. Notably, no specific distribution of *BRCA1/2* variants was observed regarding triple negative breast cancer (TNBC) or patient gender status.

**Conclusions:**

In this first genetic profiling of BC in Mauritania, we identified a substantial number of *BRCA1/2* pathogenic variants. This finding could be important in the future diagnosis and prevention policy of hereditary BC in Mauritania.

## Background

Breast cancer (BC) is the second cause of death by cancer in women [1]. Screening for inherited variants in patients with BC has significantly increased over the past 30 years. About 20% of all BC cases described worldwide have a genetic origin with a large heterogeneity in the percentage of pathogenic variants (PVs) [2–4].

Somatic variants represent the most common cause of cancer while germline variants accounts for approximately 5% of BC. Although two-thirds of these variants were found in *BRCA1/2*, other genes such as *ATM*, *PALB2*, *CHEK2*, *PTEN* and *TP53* have been also reported in hereditary BC, ovarian cancer (OC), and pancreatic cancer (PC) [5, 6]. Using NGS-based multi-gene panel testing, many cases with strong personal and/or family history of cancer were indeed found to be *BRCA1/2*-wild-type. For instance, a significant proportion of 15.1% of BC, OC, or PC germline pathogenic variants was observed in susceptibility genes other than *BRCA1/A2* among bilateral BC patients and therefore would have been over looked [7, 8].

The major risk of developing BC due to these variants and the large benefit of their early detection have made genetic screening for hereditary BC recommended to patients with family history in many countries even though the implication of these PV variants in defining the cancer clinical and phenotypic features remained unanswered [9]. Due to its comprehensive genomic coverage and higher sensitivity, next generation sequencing (NGS) has become an essential tool in tumor profiling [10]. However, this technology remained poorly used in African countries mainly due to its elevated running cost [11–13].

In this study, using NGS methodology, we reported the first *BRCA1/2* mutational profile of a BC patients’ cohort in Mauritania and assessed the relevance of detected variants to the carriers’ demographic and clinical characteristics.

## Patients and methods

### Patients

In this study, 137 Mauritanian BC patients (132 women and 5 men) whose demographic and clinical data were complete and available to participate to the genetic screening were selected if they met one of the following criteria:

- Age below 45 years when diagnosed with BC
- Bilateral BC
- BC detected in two or more relatives (first or second degree) in the family
- BC in men
- Triple negative breast cancer (TNBC) diagnosed before the age of 60 years.

Demographic and clinical characteristics of recruited subjects included age at diagnosis, family history of the disease, cancer staging and histological grading. Immunohistochemical staining (IHC), when accessible, was carried out on patient tissue samples embedded in paraffin blocks. Grading (from T0 to T4) was performed according to the American joint committee on cancer/ /Union for international cancer control (AJCC /UICC) systems. Evaluation of cancer stage (from 0 to 4) used TNM staging. TNBC patients were referred to subjects with IHC slides showing no antibody staining or a tumor cells fluorescence less than 1% for, concomitantly, receptors of estrogen (ER), progesterone (PR) and hormone epidermal growth factor receptor 2 (HER-2).

#### BRCA1 and BRACA2 molecular screening

Extraction of DNA from peripheral blood collected in EDTA tubes was carried out using QIAampDNA Blood Midi Kit (Qiagen, Hilden, Germany). DNA concentration and quality were assessed by NanoDrop (Jenway) spectrophotometer and agarose electrophoresis, respectively. Screening of the *BRCA1/2* variants was performed using the ONCO/Reveal™ *BRCA1/BRCA2* panel (Pillar Biosciences, Natick, MA). This technique consisted of a robust NGS assay to sequence the entire coding regions of the *BRCA1* and *BRCA2* genes plus 20 bp of flanking introns. It is based on the use of proprietary Stem-Loop Inhibition-Mediated amplification (SLIMamp®), a tiled amplicon-based library prep chemistry for efficient single-tube target enrichment. Sequencing was then performed on MiSeq platform as recommended by the manufacturer (Illumina, Inc., San Diego, CA) at Colorado sequencing center (https://thesequencingcenter.com/).

### Bioinformatics

Alignment of the sequence reads, in FastQ format, was referred to the reference human genome 19 (hg19). Variant annotations were carried out using databases including HapMap project (http://hapmap.ncbi.nlm.nih.gov/), 1000 Genome Project (http://www.1000genomes.org/), Exome Variant Server (EVS, http://evs.gs.washington.edu/EVS/), and Exome Aggregation Consortium (EXAC, http://exac.broadinstitute.org/).

DNA variants were considered as pathogenic if cited by ClinVar record or reported by in silico analysis with PolyPhen2, (http://genetics.bwh.harvard.edu/pph2/), Sorting Intolerant From Tolerant (SIFT, http://sift.jcvi.org/) or Mutation Taster (http://www.mutationtaster.org/).

### Statistics

Demographic and clinic-pathologic characteristics of patients were statistically assessed by SPSS data analysis package version 23.0 software (Chicago, Ill).

## Results

### Demographic and pathological features of the cohort

The demographic and clinico-pathological characteristics of 137 BC patients (132 women and 5 men) were shown in Table 1. Women were aged between 26 and 76 years with an mean age of 45 years at time of diagnosis of BC. Family history (patients with first- or second-degree relatives with BC) was reported in 41(30%) BC patients and consanguinity observed in 63 (46%) patients.

**Table 1:** Demographic and clinico-pathological characteristic of the study population

Parameters	Patients (137)	Percentage (%)
Total of patients	137	
Females	132	96.35
Males	5	3.65
Age at diagnosis (years)	Mean age Women 45 Men 67	
< 35	22	16.06
[35–55]	95	69.34
> 55	20	14.60
Familyhistory		
Present	41	29.93
Absent	96	70.07
Consanguinity		
Yes	63	45.99
Non	74	54.01
Ethnicity		
White Moors	73	53.28
Black Moors	45	32.85
Black Africans	19	13.87
Histological grading		
Grade I	12	8.6
Grade II	80	58.39
Grade III	45	32.85
Staging		
Stage I	2	1.46
Stage II	42	30.66
Stage III	61	44.53
Stage IV	32	23.36
Histological type		
Invasive Ductal Carcinoma (IDC)	114	83.21
Invasive Lobular Carcinoma (ILC)	12	8.75
	11	8.04
Immunohistochemistry (*N* = 96)		
TNBC	45	46.87
NTNB	51	53.13

Cells abnormality was diagnosed at poorly differentiated status (grade III) in 45 (32.8%) patients. BC was at stage III in 61 (44.5%) and metastasized (stage IV) in 32 (23.4%) patients.

Cancer stretching into breast surrounding tissues showed presence of invasive ductal carcinomas (IDC) and invasive lobular carcinomas (ILC) in 114 (83.2%) and 12 (8.8%) patients, respectively.

Among 96 patients with satisfactory immunohistochemical data, 45 (46.9%) were classified triple negative breast cancer (TNBC) as no antibody staining above cut off was detected simultaneously for *ER, PR* and *HER-2* receptors.

The five men with BC recruited had an age between 42 and 95 years. They all had unilateral (left or right) advanced BC with IDC in 4 of them. Only one male was TNBC.

### BRCA1 and BRCA2 sequence variants in the study population

**Fig. 1:**
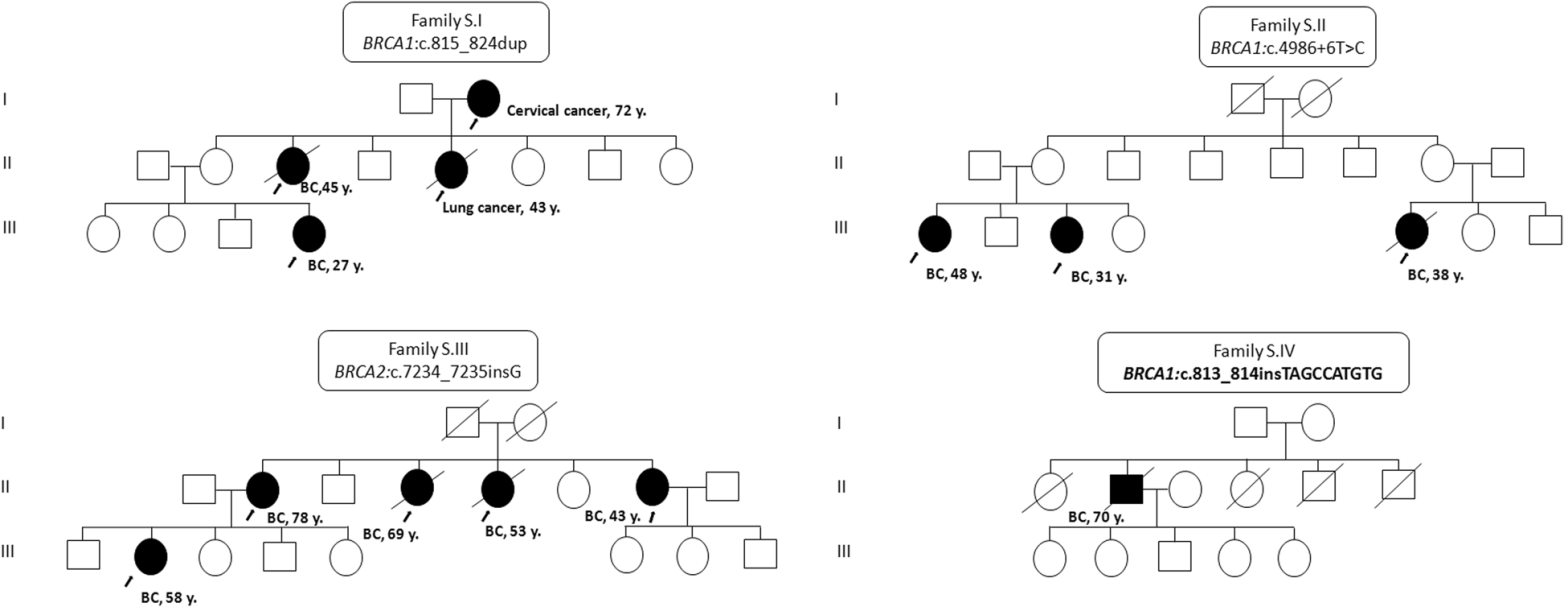
Examples of pedigrees with patients carrying variants in *BRCA1* and *BRCA2* genes in the Mauritanian population

*BRCA1/2* NGS-based screening of the cohort identified sixteen pathogenic (PV) or likely pathogenic variants (PLPVs) (8 in *BRCA1* and 8 in *BRCA2*) observed in 38 patients (28.8% of total BC patients) (Table 2). PV/LPV variants were carried by 11 different families among 41 enrolled patients with family history (1st or 2nd degree affected) of BC recruited. Pedigrees of the most prevalent pathogenic variants were presented in Fig. 1. Among the PV/LPV variants, we had seven missense, four insertions, four deletions and one splice site variants. The two predominant variants were frameshift (c.815_824dup) in exon 10 and splice site variant (c.4986 + 6 T > C) in intron 15, found in 13 and 7 BC patients, respectively. Both changes were located in *BRCA1* gene. Frameshift variants (c.7234_7235insG and c.6280_6286del) of *BRCA2* gene were identified in 4 and 2 patients, respectively.

**Table 2:** List of pathogenic and likely pathogenic *BRCA1* and *BRCA2* genes variants found in Mauritanian hereditary BC patients

Gene	Chromosome position	Mutation type	Database ID	Gene location	nucleotide change	protein change	Clinicalsignificance	Number of carriers
BRCA1	chr17:41222939–41,222,939	Intron	rs80358086	Intronic	c.4986 + 6 T > C		pathogenic	7
BRCA1	chr17:41276061–41,276,061	Missense	rs80356929	Exon2	c.53 T > C	p.Met18Thr	likely_pathogenic	1
BRCA1	chr17:41267755–41,267,755	Missense	rs80357276	Exon 3	c.122A > T	p.His41Leu	likely_pathogenic	1
BRCA2	**chr13:32893402–32,893,402**	**frameshift**	**Unreported**	**Exon 3**	**c.256del**	**p.Leu86Ter**	**Pathogenic**	1
BRCA2	**chr13:32911384–32,911,385**	**frameshift**	**Unreported**	**Exon 3**	**c.2892_2893insC**	**p.Met965HisfsTer17**	**Pathogenic**	1
BRCA2	chr13:32914767–32,914,773	frameshift	rs80359572	Exon 3	c.6280_6286del	p.Tyr2094LeufsTer23	pathogenic	2
BRCA2	chr13:32929224–32,929,225	frameshift	rs397507906	Exon 3	c.7234_7235insG	p.Thr2412SerfsTer2	pathogenic	4
BRCA2	chr13:32953609–32,953,609	stop_gained	rs886040799	Exon 3	c.8910G > A	p.Trp2970Ter	pathogenic	1
BRCA2	chr13:32953902–32,953,902	stop_gained	rs80359148	Exon 3	c.8969G > A	p.Trp2990Ter	pathogenic	1
BRCA1	chr17:41267746–41,267,746	Missense	See in ClinVar	Exon 4	c.131G > C	p.Cys44Ser	Pathogenic	1
BRCA1	chr17:41215374–41,215,374	frameshift	rs80357553	Exon 4	c.5169del	p.Glu1725LysfsTer5	Pathogenic	1
BRCA1	chr17:41244539–41,244,540	frameshift	rs80357617	Exon4	c.3008_3009del	p.Phe1003Ter	pathogenic	1
BRCA1	**chr17:41246734–41,246,735**	**stop_gained**	**Unreported**	**Exon 4**	**c.813_814insTAGCCATGTG**	**p.Glu272Ter**	**Pathogenic**	1
BRCA1	chr17:41246723–41,246,724	frameshift	rs387906563	Exon 10	c.815_824dup	p.Thr276Alafs*14	pathogenic	13
BRCA2	chr13:32914292–32,914,292	stop_gained	rs886040610	Exon 11	c.5800C > T	p.Gln1934Ter	pathogenic	1
BRCA2	chr13:32914617–32,914,617	Missense	rs80358852	Exon 11	c.6125A > G	p.Gln2042Arg	Conflicting of pathogenicity	1

*Novel mutations (3 unreported) are shown in bold

Interestingly, we identified four variants never reported before (Tables 2 and 3): One (c.813_814insTAGCCATGTG) in *BRCA1* and three (c.256del, c.2892_2893insC and c.10247A > G) in *BRCA2*. Three of these novel variants were predicted pathogenic (Table 2).

**Table 3:** List of non-pathogenic *BRCA1* and *BRCA2* genes variants found in Mauritanian hereditary BC patients

Gene	Chromosome position	Mutation type	Database ID	Gene location	nucleotide change	protein change	Clinicalsignificance	Number of carriers
BRCA2	chr13:32893271–32,893,271	Missense	rs4987046	Exon 3	c.125A > G	p.Tyr42Cys	benign	1
**BRCA2**	**chr13:32972897–32,972,897**	**Missense**	**Unreported**	**Exon 3**	**c.10247A > G**	**p.Lys3416Arg**	**benign**	**1**
BRCA1	chr17:41246481–41,246,481	Missense	rs1799950	Exon 9	c.1067A > G	p.Gln356Arg	benign	1
BRCA1	chr17:41246411–41,246,411	Missense	rs56128296	Exon 9	c.1137 T > G	p.Ile379Met	benign	1
BRCA1	chr17:41245471–41,245,471	Missense	rs4986850	Exon 9	c.2077G > A	p.Asp693Asn	benign	5
BRCA1	chr17:41256155–41,256,155	Missense	rs55971303	Exon 10	c.425C > A	p.Pro142His	benign	1
BRCA2	chr13:32906480–32,906,480	Missense	rs766173	Exon 10	c.865A > C	p.Asn289His	benign	1
BRCA1	chr17:41245090–41,245,090	Missense	rs56082113	Exon 10	c.2458A > G	p.Lys820Glu	benign	2
BRCA1	chr17:41244429–41,244,429	Missense	rs4986852	Exon 10	c.3119G > A	p.Ser1040Asn	benign	2
BRCA1	chr17:41244936–41,244,936	Missense	rs799917	Exon11	c.2612C > T	p.Pro871Leu	benign	70
BRCA2	chr13:32911278–32,911,278	Missense	rs2227943	Exon 11	c.2786 T > C	p.Leu929Ser	benign	1
BRCA2	chr13:32912679–32,912,679	Missense	rs55969723	Exon 11	c.4187A > G	p.Gln1396Arg	benign	1
BRCA2	chr13:32914132–32,914,132	Missense	rs11571657	Exon 11	c.5640 T > G	p.Asn1880Lys	benign	1
BRCA2	chr13:32914196–32,914,196	Missense	rs4987048	Exon 11	c.5704G > A	p.Asp1902Asn	benign	1
BRCA2	chr13:32914712–32,914,712	Missense	rs34309943	Exon 11	c.6220C > A	p.His2074Asn	benign	1
BRCA1	chr17:41226423–41,226,423	Missense	rs55815649	Exon 13	c.4600G > A	p.Val1534Met	benign	1
BRCA2	chr13:32929309–32,929,309	Missense	rs4986860	Exon 14	c.7319A > G	p.His2440Arg	benign	1
BRCA2	chr13:32953529–32,953,529	Missense	rs4987047	Exon 22	c.8830A > T	p.Ile2944Phe	benign	1
BRCA2	chr13:32972884–32,972,884	Missense	rs1801426	Exon 27	c.10234A > G	p.Ile3412Val	benign	3

*Novel mutations (1 unreported) are shown in bold

Twelve of the 42 TNBC patients carried pathogenic variants: 7 in *BRCA1* and 5 in *BRCA2*. Nineteen non pathogenic variants (8 in *BRCA1* and 11 in *BRCA2*) were detected in 96 BC patients (73.8%) (Table 3).

**Fig. 2:**
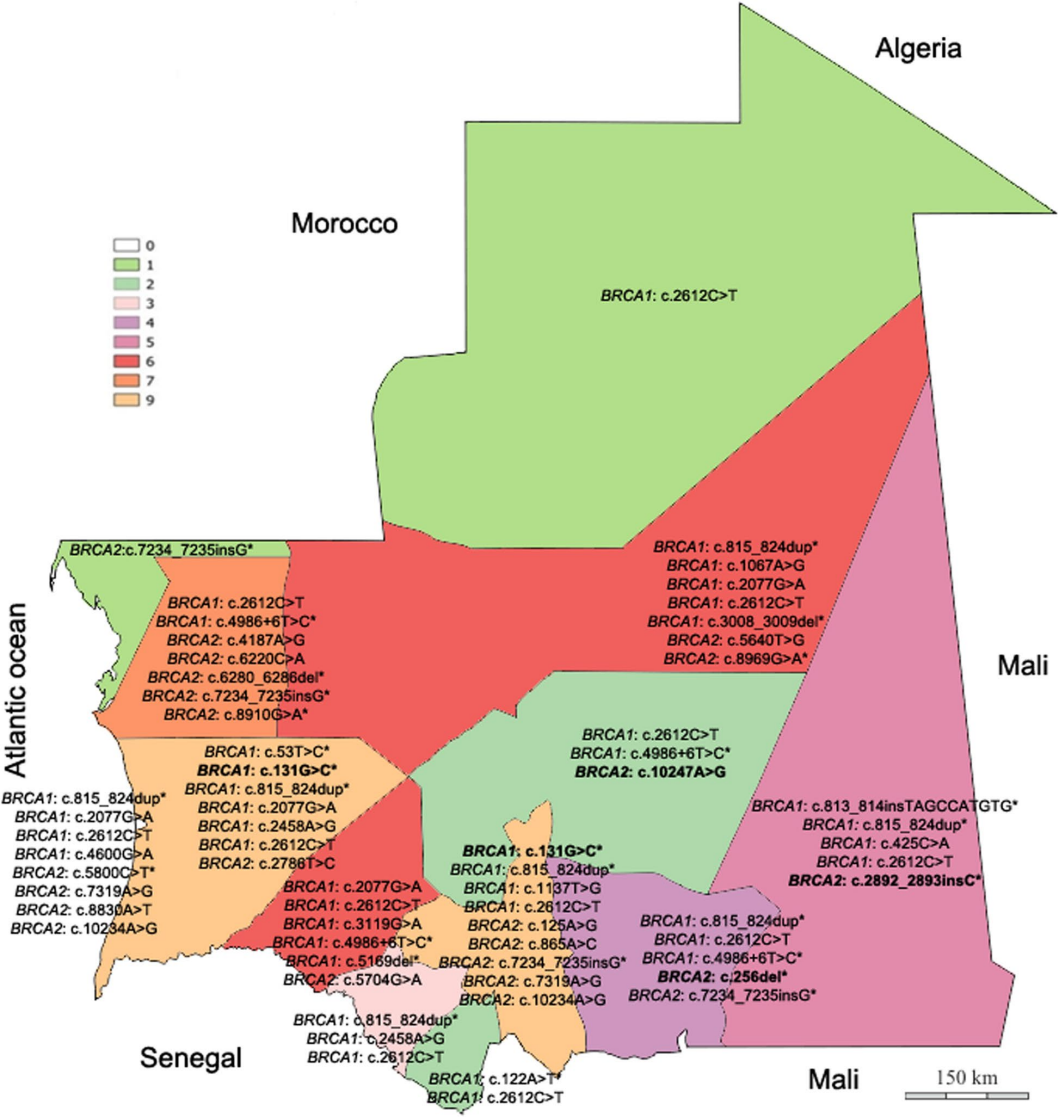
Geographic distribution of reported *BRCA1/2* variants in Mauritania

Carriers of all variants originated from different regions of the country (Fig. 2).

## Discussion

Although poor socioeconomic conditions and reduced access to adequate healthcare may have been determinant in BC development and outcome among our cohort, genetic origin was also likely giving the high rate of consanguinity (46%), the early average age of women (45 years) and advanced phase of BC at diagnosis observed in our cohort. Indeed, numerous data showed a higher incidence of common adult diseases such as hypertension, diabetes mellitus or cancer in patients from consanguineous marriages [14]. Reports also indicated that close-kin marriage continued to be preferential in North and sub-Saharan Africa with a prevalence of marriages between couples related as second cousins or closer exceeding 40% in countries including Mauritania [15, 16].

Gene predisposition has been suggested in the younger median age at BC diagnosis among Eastern Mediterranean women compared to women in Western European countries [17, 18]. An equivalent early age at BC detection (47 years) was also observed in Sub-Saharan and British black women while most Caucasian women had a BC onset at 67 years [19–22].

In this first study providing molecular data on BC in our country, among the 137 BC patients recruited at the only state cancer referring center, 38 (27.7%) carried a *BRCA1/2* pathogenic or likely pathogenic variant. In neighboring North African region, the frequency of *BRCA1/2* pathogenic variants in Moroccan population varied from 16.7 to 31.6% [23]. In Tunisia, 25% of hereditary BC patients carried a *BRCA1/2* gene pathogenic variant [24]. The *BRCA1/2* variant ratio we found (1:1.18) was also consistent with the fraction reported by these different studies which confirmed the significant contribution of *BRCA1/2* germline variant in BC risk among North African populations [25, 26]. Although we did not perform functional validation of the *BRCA1/2* deleterious variants identified in our cohort, most common PV/LPVs we found were already reported as associated with BC. Indeed (c.815_824dup p.Thr276Alafs*14) (found here in 34.2% of patients), was diagnosed in 15 of 27 (55.5%) patients of hereditary BC cases in neighboring Senegal with a founder effect in Afro-American patients [27]. This *BRCA1* duplication leading to a premature stop codon was revealed as pathogenic variant by ClinVar here and in other studies [27, 28]. It was also submitted by HGSC-CL as pathogenic germline variant using clinical testing [29, 30]. The relatively high and concordant frequency in our cohort and in Senegal further highlights the pathogenic character of this variant in BC assessment and prevention in West African families [27, 31–33].

The second pathogenic variant (c.4986 + 6 T > C) in *BRCA1* carried by seven of our BC patients has also been detected in multiple African patients with an early-onset of BC [34, 35]. This splicing site variant is located in highly conserved human genome region and seems to activate a cryptic splice donor site which alters the reading frame resulting in absent or truncated protein [36, 37]. It was proposed by ClinVar as pathogenic germline allele by numerous submitters [35, 38].

We also found that among the 12 TNBC patients carrying a pathogenic or likely pathogenic variant, 7 had a *BRCA1* variant which substantiates data suggesting that patients with *BRCA1* variants were more likely to have TNBC than those with *BRCA2* variants [39, 40].

Due to the limited number of BC patients here, we could not conclude on the association of the pathogenic variant frequency and the BC phenotypic features (molecular tumor subtypes) as investigated elsewhere [40]. For instance, *BRCA1*-633delC was detected with relatively higher prevalence in patients with TNBC, whereas *BRCA2*-1466delT was found mainly in Luminal B tumors, but not in TNBC patient [40].

Our study also identified three novel predicted pathogenic *BRCA1/2* variants never reported before. These variants could be specific to our population. Territorial prevalence has for instance reported among families with hereditary breast and ovarian cancer in families from southern Italy with a higher prevalence of PVs in Sicilian population [41].

One limitation of this single gene testing was that only *BRCA1/2* was explored in this cohort while a NGS-based multi-gene panel testing could have revealed more potentially BC pathogenic variants. It was thus showed that 19 out of 53 positively tested bilateral BC patients harbored a germline PVs in a known (no-*BRCA)* BC susceptibility gene which clearly support the inclusion of multi-gene panel with high or intermediate penetrant BC genetic predisposition [7, 8]. Although highly pertinent, we could not currently perform such a study giving the high cost of this profiling.

## Conclusions

Our study gave the first data on *BRCA1/2* alterations likely underlying hereditary BC in Mauritania using a powerful NGS based screening.

A multi-gene panel testing of all BC patients followed by Sanger sequencing confirmation should avoid underestimation of affected patients and pave way to more cancer associated PV/LPV variants identification in hereditary tumor surveillance and targeted therapy choice.

Most of PV/LPVs variants identified in our cohort were previously reported in other African populations geographically remote and culturally apparently not related. This BC heterogeneous heredity confirmed the complex genetic structure of African populations shaped by successive voluntary or forced migrations, integrations and assimilations over many centuries.

## Data Availability

The datasets on unpublished pathogenic variants generated and/or analyzed during the current study are not publicly available as the study is still ongoing on other breast cancer genes and other cancers than breast cancer but are available from the corresponding author on reasonable request. The datasets of already published pathogenic variants study are available in [ClinVar] repository, via following WEB links-. https://www-ncbi-nlm-nih-gov.proxy.insermbiblio.inist.fr/clinvar/docs/submit/ https://urldefense.com/v3/ https://ftp-ncbi-nlm-nih-gov.proxy.insermbiblio.inist.fr/pub/clinvar/submission_templates/SubmissionTemplateLite.xlsx__;!!NLFGqXoFfo8MMQ!oy4EkQpiIJT0YVAimoRVIjQQrqMoJViV-xEpFEQKcqcfhfbZzOIAogOGqDRGJcJAj4Npv-u8tpFqoQ3rTV5bOOMpbTdw%2 https://submit-ncbi-nlm-nih-gov.proxy.insermbiblio.inist.fr/clinvar/

## Abbreviations

BC: Breast cancer
BRCA: Breast cancer gene
PV: Pathogenic variant
LPV: Likely pathogenic variant
NGS: Next generation sequencing
TNBC: Triple negative breast cancer
OC: Ovarian cancer
PC: Pancreatic cancer
IHC: Immunohistochemical staining
IDC: Invasive ductal carcinomas
ILC: Invasive lobular carcinomas
